# Dietary choline intake and its association with asthma: A study based on the National Health and Nutrition Examination Survey database

**DOI:** 10.1002/clt2.12359

**Published:** 2024-06-11

**Authors:** Jiaqiang Shi, Yuming Lin, Yingxiu Jiang, Guoguo Qiu, Fanghua Jian, Wei Lin, Shihao Zhang

**Affiliations:** ^1^ Department of Pediatrics Longyan First Hospital of Fujian Medical University Longyan Fujian Province China; ^2^ Minxi Vocational College (Fujian) Longyan Fujian Province China; ^3^ Department of Respiratory and Critical Care Medicine Ganzhou People's Hospital Zhangzhou Jiangxi Province China

**Keywords:** asthma, dietary choline intake, logistic regression, methyl donors, NHANES

## Abstract

**Objective:**

This work endeavored to examine the correlation between dietary choline intake and the odds of asthma, utilizing data from the National Health and Nutrition Examination Survey (NHANES).

**Methods:**

Aggregated data from seven cycles (2005–2018) in the NHANES database were utilized. The independent variable was dietary choline intake, and the dependent variable was asthma. The weighted logistic regression method was used to construct a model reflecting the relationship between these two factors. This work employed stratified analysis without adjusting for confounding factors and subgroup analysis with adjusted confounding factors to mine the association between dietary choline intake and asthma. Additionally, restricted cubic spline analysis examined nonlinear associations of the two in age subgroups.

**Results:**

Forty five thousand and seven hundreds ninety seven samples were included here. The model indicating the relationship between dietary choline intake and asthma was constructed (OR: 0.86, 95% CI: 0.79–0.93, *p* < 0.001). Stratified analysis indicated that the interaction terms of age (*p* < 0.001) and body mass index (BMI) (*p* = 0.002) with dietary choline intake significantly influenced the relationship model. In the adjusted models, accounting for demographic characteristics, poverty impact ratio, BMI, exposure to environmental tobacco smoke, and total energy intake, an increase in dietary choline intake significantly reduced the odds of asthma (OR: 0.79, 95% CI: 0.72–0.88, *p* < 0.001). Subgroup analyses based on age and BMI revealed a significant negative correlation between dietary choline intake and the odds of asthma in the adult population (OR: 0.76, 95% CI: 0.67–0.86, *p* < 0.001), as well as in individuals with a BMI between 25 and 30 kg/m^2^ (OR: 0.79, 95% CI: 0.63–0.99, *p* = 0.042), and those with a BMI >30 kg/m^2^ (OR: 0.73, 95% CI: 0.60–0.89, *p* = 0.002).

**Conclusion:**

Dietary choline intake was significantly inversely correlated with asthma prevalence, especially in adults and overweight/obese individuals, suggesting that increasing choline intake may reduce asthma risk. Further research is needed to explore this relationship and provide tailored dietary recommendations for different age and BMI groups to enhance asthma prevention and management.

## 1 INTRODUCTION

In 2023, the Global Initiative for Asthma (GINA) presented the “Global Strategy for Asthma Management and Prevention,” defining asthma as a condition characterized by difficulty exhaling air from the lungs due to bronchoconstriction (narrowing of the airways), thickening of the airway walls, and increased mucus production.^1^ It is characterized by fluctuating respiratory symptoms, including wheezing, breathlessness, chest tightness, and cough, which exhibit variability in both time and intensity. These symptoms are accompanied by reversible constriction of airflow.^2^ Asthma can manifest at any age, and its symptoms can range from absent or mild to severe.^3^ As of 2021, according to the latest report from the Centers for Disease Control and Prevention, an estimated 24.96 million individuals in the U.S. are affected by asthma, with 39.4% of them having suffered asthma attacks in the past 12 months, resulting in 10.6% of patient deaths.^4^ Asthma not only profoundly affects physical and mental well‐being but also hampers learning efficiency, limits physical activity, and decreases overall quality of life.^5^ The socioeconomic impact is also substantial. Statistics indicate an annual loss exceeding 80 billion dollars due to factors such as overall mortality and medical costs.^6^ Therefore, there is an urgent need to comprehend the contributing factors to asthma, as this knowledge can inform the development of effective primary public health and pharmacological prevention measures aimed at reducing asthma prevalence.

Some viewpoints suggest that one of the mechanisms through which diet influences asthma and allergic susceptibility is via DNA methylation, an epigenetic mechanism that leads to heritable alterations in gene expression without modifying the primary DNA sequence.^7^, ^8^ Choline (2‐hydroxyethyl‐trimethyl‐ammonium salt; molecular weight of 104 g/mol), as one of the crucial dietary methyl donors in the human body, influences DNA methylation by participating in the one‐carbon metabolism pathway.^9^ Dietary choline exists in various forms, including both water‐soluble forms (such as free choline, phosphatidylcholine, and glycerophosphocholine) and fat‐soluble forms (such as sphingomyelin and phosphatidylcholine).^10^ The U.S. Department of Agriculture (USDA) estimates choline intake in various foods,^11^ including sources from animals (such as liver, eggs, pork, and beef) and plants (such as soybeans, peanuts, broccoli, and cauliflower). Previous animal experiments have shown that exposure to diets rich in methyl donors (including folate, choline, and vitamin B12) increases airway reactivity in maternal and offspring mice, leading to negative regulation of allergic airway diseases.^12^ Moreover, studies have reported a linkage between high dietary choline intake and lower levels of inflammatory markers.^13^ Choline therapy has been shown to modulate immune inflammation and repress oxidative stress in asthma patients, holding promise as an adjunctive therapy for asthma.^14^ While these findings are intriguing, data specifically focusing on the effects of individual methyl donors, such as choline, are lacking, and clinical research concerning the linkage between dietary choline intake and asthma incidence remains absent. Hence, the connection between dietary choline and asthma in human subjects remains ambiguous, necessitating further inquiries to clarify the involvement of choline in the pathogenesis of asthma.

Based on the above research background, we posited a potential link between dietary choline intake and the occurrence of asthma. To examine the validity of our hypothesis, we harnessed data sourced from the National Health and Nutrition Examination Survey (NHANES) database, encompassing a time frame spanning seven cycles spanning from 2005 to 2018. We conducted an association analysis between dietary choline intake and asthma using a weighted logistic regression model. Additionally, we explored the impact of dietary choline intake on asthma incidence under various conditions, including age, race, gender, poverty impact ratio (PIR), body mass index (BMI), total energy intake, and exposure to environmental tobacco smoke (ETS) through stratified analyses.

## 2 METHODS

### 2.1 Data source and study population

This study is a cross‐sectional investigation utilizing data sourced from the NHANES database. NHANES is a stratified multistage study conducted by the National Center for Health Statistics (NCHS) in the U.S.^15^ This database, accessible free of charge, features a comprehensive assessment of the health and nutritional status of the U.S. population through a combination of interviews, physical examinations, and laboratory testing. The data collection process for NHANES has received approval from the NCHS Institutional Review Board, with participants offering informed consent.

**FIGURE 1:**
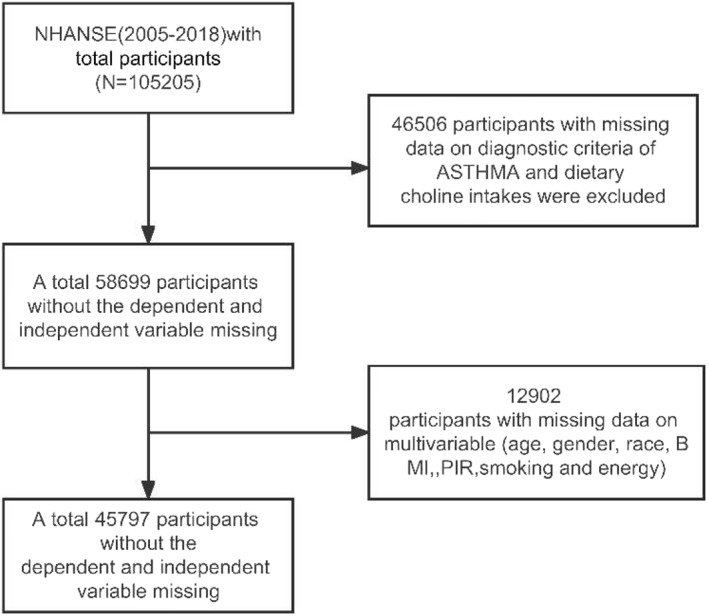
Sample selection flowchart for National Health and Nutrition Examination Survey 2005–2018.

A total of 105,205 participants from seven consecutive NHANES cycles spanning from 2005 to 2018 were initially selected for this study. Dietary choline intake serves as the exposure variable, while the occurrence of asthma is the observed outcome. To ensure the integrity of the information for each sample, individuals were excluded based on certain criteria. Specifically, 46,506 respondents lacking information on asthma diagnosis or dietary choline intake were excluded, along with 12,902 respondents with missing covariates (age, gender, race, BMI, PIR, ETS, and total energy intake). Ultimately, 45,797 eligible respondents were included in the study sample. The detailed participant selection process is illustrated in Figure 1.

### 2.2 Dietary choline intake assessment

Data collection on dietary choline intake in NHANES is based on two 24‐h dietary recall interviews conducted for all participants. The first dietary recall is conducted at the Mobile Examination Center, with a follow‐up telephone interview 3–10 days later for the second recall, gathering information on all foods and beverages consumed. Subsequently, interviewers use a specific coding system (the Food Code System of the USDA to link each reported food and beverage to corresponding entries in the Food Composition Database.^16^ The intake of dietary choline for each food is calculated by multiplying its intake by the choline content listed in the Food Composition Database. The choline intake from all foods is then summed to obtain the total dietary choline intake for the participants during the specified time period.^17^ To approximate a normal distribution of the data, the dietary choline intake is log‐transformed, and subsequent analyses utilize the transformed values. Weight selection follows the criteria outlined as follows: (a) In the absence of a second dietary recall weight, the first dietary recall weight (WTDRD1) is used. (b) If a second dietary recall weight exists (including whether the first dietary recall weight is present), the second dietary recall weight (WTDRD2) is utilized.

### 2.3 Asthma definition

In this study, participants who responded affirmatively to the question in the NHANES data collection questionnaire regarding whether they had been diagnosed with asthma were categorized as having asthma.

### 2.4 Covariates

For this project, potential covariates and confounding factors were identified as gender, age, race, PIR, BMI, exposure to ETS, and total energy intake. Participants were categorized as either children and adolescents (<18 years old) or adults (≥18 years old) at the time of screening. Gender was classified as male or female. Race was categorized into Mexican‐American, Hispanic, non‐Hispanic White, non‐Hispanic Black, and other races. BMI was grouped according to ≤25 kg/m^2^, 25–30 kg/m^2^, or >30 kg/m^2^. PIR was classified as ≤1.3, 1.3–3.5, or >3.5.^18^ Exposure to ETS was determined based on serum cotinine concentration, with a threshold set at 14 ng/mL for defining exposure status.^18^

Total energy intake data were derived from the 24‐h dietary recall surveys within the NHANES database and processed in the same manner as dietary choline intake data.

### 2.5 Statistical analysis

The statistical analysis for this study was performed using the R programming language (v4.2.1). The creation of baseline tables was executed using the “tableone” package, while data manipulation and analysis were conducted using the “survey” package. Initially, baseline tables were generated to display the distribution of overall characteristics among respondents. These tables were then stratified based on the presence or absence of asthma. Categorical variables were presented with sample size (*n*) and corresponding proportions, while continuous variables were displayed as mean values and standard deviations. Sample size (*n*) was not subject to weighting adjustments, while proportions, mean values, and standard deviations were adjusted for weighting. Subsequently, a weighted logistic regression model was constructed to establish the relationship between dietary choline intake and asthma. The model was further stratified based on factors such as gender, age, race, PIR, BMI, ETS, and total energy intake. *p*‐values for interaction terms were determined utilizing the chi‐squared test. Furthermore, the relationship between dietary choline intake and asthma was modeled while adjusting for confounding factors. Three models were constructed: Crude (unadjusted for confounding factors), Model I (adjusted for age, race, gender, and PIR), and Model II (adjusted for age, race, gender, PIR, BMI, total energy intake, and ETS). Subgroup analyses were performed for age and BMI factors within the aforementioned models. In addition, the restricted cubic spline (RCS) method was utilized to explore potential non‐linear associations between dietary choline intake and asthma within age‐stratified groups. Results were presented as Odds Ratios (OR) along with 95% confidence intervals (CI), and statistical significance was determined at a threshold of *p* < 0.05.

## 3 RESULTS

### 3.1 Baseline characteristics of participants

The distribution of baseline characteristics among the 45,797 total samples is presented in Table 1. The male proportion (49.2%) was slightly lower than the female proportion (50.8%). The majority of participants were adults (81.1%), with an average age of 45.92 ± 17.35. Children and adolescents comprised 18.9% of the sample, with an average age of 10.58 ± 4.22. Non‐Hispanic Whites made up 65.2% of the samples, and 79.7% were exposed to ETS. The mean dietary choline intake was 5.64 ± 0.51 mg. The mean total energy intake was 7.56 ± 0.4 kcal.

**TABLE 1:** Distribution of overall characteristics of the sample.

Characteristics	*N*(%)/mean ± SD
Overall	45,797
Gender	
Female	23,147 (50.8)
Male	22,650 (49.2)
Age	
≥18	32,243(81.1)
<18	13,554 (18.9)
Race	
Mexican American	8527 (10.1)
Other Hispanic	4218 (5.6)
Non‐Hispanic White	18,071 (65.2)
Non‐Hispanic Black	10,139 (11.3)
Other race	4842 (7.8)
PIR	
≤1.3	16,349 (24.5)
1.3–3.50	17,017 (35.6)
>3.5	12,431 (39.9)
BMI(kg/m^2^)	
≤25	20,856 (41.0)
25–30	11,931 (28.1)
>30	13,010 (30.9)
ETS	
Yes	37,639 (79.7)
No	8158 (20.3)
Asthma	
No	38,626 (84.6)
Yes	7171 (15.4)
Choline (mg)	317.71 (160.43)
Log‐formed choline (mg)	5.64 (0.51)
Total energy (kcal)	2079.40 (827.98)
Log‐formed total energy (kcal)	7.56 (0.40)

*Note*: *n* is not weighted, *n* (%), mean and sd are weighted adjusted.

Abbreviations: BMI, body mass index; ETS, environmental tobacco smoke; PIR, poverty impact ratio.

The distribution of characteristics categorized by asthma status is outlined in Table 2. The asthma prevalence was 15.7% (7171/45,797). In the diseased group, the average age for adults was 43.54 ± 17.36, while for children and adolescents, it was 11.33 ± 3.92. In the non‐diseased group, the comparison between adults and children/adolescents yielded ages of 46.33 ± 17.32 and 10.43 ± 4.26, respectively. Significant differences (*p* < 0.001) were observed between individuals with and without asthma in terms of gender, age, race, PIR, BMI, ETS exposure, and dietary choline intake. Specifically, individuals with asthma exhibited significantly lower dietary choline intake (5.61 ± 0.51 mg) compared with those without asthma (5.65 ± 0.51 mg) (*p* < 0.001).

**TABLE 2:** Characteristic distribution of asthma patients and non‐asthma individuals.

Characteristics	Non‐asthma	Asthma	*p* value
Overall	38,626	7171	
Gender			<0.001*
Female	19,379 (49.9)	3768 (55.7)	
Male	19,247 (50.1)	3403 (44.3)	
Age			<0.001*
≥18	27,485 (81.5)	4758 (78.4)	
<18	11,141 (18.5)	2413 (21.6)	
Race			<0.001*
Mexican American	7605 (10.6)	922 (7.4)	
Other Hispanic	3514 (5.6)	704 (5.6)	
Non‐Hispanic White	15,146 (65.2)	2925 (64.9)	
Non‐Hispanic Black	8230 (10.8)	1909 (14.2)	
Other race	4131 (7.8)	711 (7.8)	
PIR			<0.001*
≤1.3	13,479 (23.5)	2870 (29.7)	
1.3–3.50	14,516 (36.0)	2501 (33.5)	
>3.5	10,631 (40.4)	1800 (36.8)	
BMI(kg/m^2^)			<0.001*
≤25	17,765 (41.3)	3091 (39.2)	
25–30	10,272 (28.7)	1659 (25.2)	
>30	10,589 (30.0)	2421 (35.7)	
ETS			<0.001*
Yes	31,957 (80.3)	5682 (76.5)	
No	6669 (19.7)	1489 (23.5)	
Choline (mg)	319.24 (159.73)	309.26 (164.03)	0.004*
Log‐formed choline (mg)	**5.65 (0.51)**	**5.61 (0.51)**	<0.001*
Total energy (kcal)	2075.70 (819.07)	2099.78 (875.23)	0.250
Log‐formed total energy (kcal)	7.56 (0.40)	7.57 (0.41)	0.638

*Note*: *n* is not weighted, *n* (%), mean and sd are weighted adjusted. Data in bold represent statistically significant differences at *p*＜0.001.

Abbreviations: ETS, exposure to environmental tobacco smoke; PIR, poverty impact ratio.

*p < 0.05.

### 3.2 Linkage between dietary choline intake and asthma

Subsequently, we constructed a relationship model for dietary choline intake and asthma using the weighted logistic regression method, as presented in Table 3. The initial results, without adjusting for any confounding factors, indicated that dietary choline intake served as a protective factor against asthma (OR: 0.86, 95% CI: 0.79–0.93, *p* < 0.001). With increasing dietary choline intake, the odds of asthma incidence decreased.

**TABLE 3:** The logistic regression model of dietary choline intake and asthma.

Characteristic	OR	95% CI	*p*‐value
Choline (mg/dL)	0.86	(0.79, 0.93)	<0.001*

*Note*: Data in bold represent statistically significant differences at *p* < 0.001.

Abbreviation: OR, Odds Ratios.

*p < 0.05.

### 3.3 Subgroup analysis

As shown in Table 4, a stratified analysis was performed on the weighted logistic model for the relationship between dietary choline intake and asthma, without adjusting for confounding factors. The results revealed a significant impact of dietary choline intake on reducing the odds of asthma in the adult population (age ≥18 years) (OR: 0.82, 95% CI: 0.74–0.90, *p* < 0.001). However, among the children and adolescents (age <18 years), increased dietary choline intake was notably linked with elevated odds of asthma (OR: 1.21, 95% CI: 1.04–1.42, *p* = 0.015). Among individuals with a BMI ranging from 25 to 30 kg/m^2^ (OR: 0.8, 95% CI: 0.68–0.94, *p* = 0.006) as well as those with a BMI exceeding 30 kg/m^2^ (OR: 0.75, 95% CI: 0.66–0.86, *p* < 0.001), a noteworthy reduction in the likelihood of asthma incidence was observed with increased dietary choline intake. Furthermore, the interaction terms between age (*p* < 0.001) and BMI (*p* = 0.002) with dietary choline intake remained statistically significant in the model after adjusting for all confounding factors.

**TABLE 4:** Multivariable stratified model of the relationship between dietary choline intake and asthma.

Participants	OR	95% CI	*p*‐value	*p* for interaction
Gender				0.797
Female	0.91	(0.81–1.03)	0.14	
Male	0.91	(0.81–1.02)	0.086	
Age				<0.001*
≥18	**0.82**	**(0.74–0.90)**	**<0.001**	
<18	1.21	(1.04–1.42)	0.015	
Race				0.079
Mexican American	0.69	(0.57–0.84)	<0.001	
Other Hispanic	0.85	(0.69–1.06)	0.14	
Non‐Hispanic White	0.86	(0.76–0.98)	0.02	
Non‐Hispanic Black	1.05	(0.92–1.20)	0.4	
Other race	0.84	(0.67–1.04)	0.11	
PIR				0.699
≤1.3	0.89	(0.79–0.99)	0.031	
1.3–3.50	0.91	(0.80–1.05)	0.2	
>3.5	0.84	(0.71–1.00)	0.047	
BMI (kg/m^2^)				0.002*
≤25	1	(0.90–1.11)	>0.9	
25–30	**0.8**	**(0.68–0.94)**	**0.006**	
>30	**0.75**	**(0.66–0.86)**	**<0.001**	
ETS				0.152
Yes	0.87	(0.79–0.96)	0.006	
No	0.81	(0.71–0.93)	0.002	

*Note*: Data in bold represent statistically significant differences at *p* < 0.05

Abbreviations: ETS, exposure to environmental tobacco smoke; PIR, poverty impact ratio.

*p < 0.05.

**FIGURE 2:**
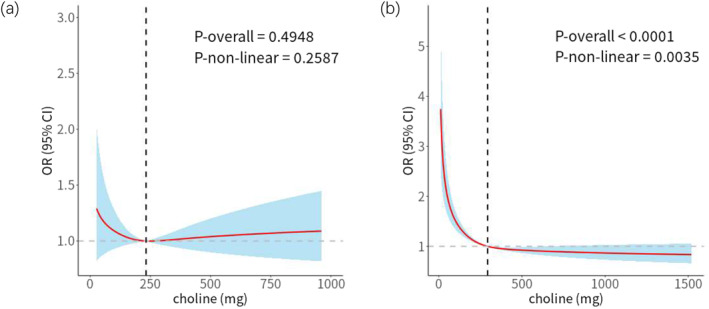
Restricted cubic spline plot of the association between dietary choline intake and increase in odds of asthma. (A) Children and adolescents; (B) Adults. Adjusted for age, race, gender, poverty impact ratio, body mass index, total energy intake, and environmental tobacco smoke.

As depicted in Table 5, different models were constructed by adjusting for various confounding factors. Both the Crude model (OR: 0.86, 95% CI: 0.79–0.93, *p* < 0.001) and Model II (OR: 0.79, 95% CI: 0.72–0.88, *p* < 0.001) demonstrated a significant reduction in the odds of asthma associated with dietary choline intake across the entire study population. Results from age‐stratified subgroup analyses indicated a notable reduction in the odds of asthma linked with dietary choline intake in both the Crude model (OR: 0.82, 95% CI: 0.74–0.90, *p* < 0.001) and Model II (OR: 0.76, 95% CI: 0.67–0.86, *p* < 0.001) for adults (age ≥18 years). Considering the substantial inter‐group differences in the age subgroup analysis, RCS analysis was performed to explore non‐linear relationships within age‐stratified groups (Figure 2). The RCS curves for children and adolescents (Figure 2A) suggested no obvious non‐linear relationship between dietary choline intake and the odds of asthma after adjusting for confounding factors (*p*‐non‐linear = 0.2587), even indicating a lack of evident correlation (*p*‐overall = 0.4948). In contrast, RCS curves for adults (Figure 2B) demonstrated an L‐shaped curve indicating a significant non‐linear relationship (*p*‐overall <0.0001, *p*‐non‐linear = 0.0035) between dietary choline intake and the odds of asthma after adjusting for confounding factors.

**TABLE 5:** Relationship models and subgroup analysis after adjustment for different confounding factors.

Participants	Models	OR	95% CI	*p*‐value
All participants	Crude	0.86	(0.79–0.93)	<0.001 *
	Model I	0.98	(0.89–1.06)	0.6
	**Model II**	**0.79**	**(0.72–0.88)**	<0.001*
Age				
≥18	**Crude**	**0.82**	**(0.74–0.90)**	<0.001*
	Model I	0.94	(0.85–1.04)	0.2
	**Model II**	**0.76**	**(0.67–0.86)**	<0.001*
<18	**Crude**	**1.21**	**(1.04–1.42)**	0.015*
	**Model I**	**1.21**	**(1.03–1.43)**	0.019*
	Model II	0.98	(0.78–1.22)	0.8
BMI				
≤25	Crude	1	(0.90–1.11)	>0.9
	Model I	1.06	(0.95–1.18)	0.3
	Model II	0.86	(0.73–1.03)	0.1
25–30	**Crude**	**0.8**	**(0.68–0.94)**	0.006 *
	Model I	0.96	(0.81–1.15)	0.7
	**Model II**	**0.79**	**(0.63–0.99)**	0.042*
>30	**Crude**	**0.75**	**(0.66–0.86)**	<0.001*
	Model I	0.92	(0.80–1.05)	0.2
	**Model II**	**0.73**	**(0.60–0.89)**	0.002*

*Note*: Crude: no adjustment; model I: adjusted for age, race, gender, and PIR; Model II: the adjustment for age, race, sex, PIR, BMI, the total energy intake and ETS. Data in bold represent statistically significant differences at *p*＜0.05.

Abbreviations: BMI, body mass index; ETS, environmental tobacco smoke; PIR, poverty impact ratio.

*p < 0.05.

Subgroup analysis based on BMI (Table 5) revealed that in individuals with a BMI between 25 and 30 kg/m^2^, both the Crude model (OR: 0.8, 95% CI: 0.68–0.94, *p* = 0.006) and Model II (OR: 0.79, 95% CI: 0.63–0.99, *p* = 0.042) demonstrated a significant reduction in the odds of asthma linked with dietary choline intake. Similarly, among individuals with a BMI >30 kg/m^2^, the Crude model (OR: 0.75, 95% CI: 0.66–0.86, *p* < 0.001) and Model II (OR: 0.73, 95% CI: 0.60–0.89, *p* = 0.002) both indicated a significant decrease in the odds of asthma due to dietary choline intake.

## 4 DISCUSSION

This work marked the inaugural cross‐sectional investigation utilizing the U.S. NHANES database to evaluate the correlation between dietary choline intake and the odds of asthma. Our findings revealed that dietary choline intake among individuals with asthma was considerably lower than that among those without asthma. Moreover, a strong and inverse correlation was confirmed between dietary choline intake and the likelihood of asthma incidence, which denoted that an elevated dietary choline intake was associated with a substantial decrease in the odds of asthma. Subsequent analyses underscored the impact of age and BMI on the relationship between dietary choline intake and the odds of asthma.

As both allergic and non‐allergic factors can trigger and exacerbate asthma, a portion of asthma occurrences can be attributed to changes in the environment and lifestyle, including dietary habits.^19^, ^20^ Prior research has identified associations between asthma or asthma symptoms and various dietary factors such as alcohol consumption,^21^ fruit and vegetable intake,^22^ dairy consumption,^23^ fish intake,^24^ and meat consumption.^25^ However, the connection between choline, a dietary nutrient found abundantly in meats (liver, poultry, fish, shellfish), eggs, and vegetables,^26^ and the occurrence and development of asthma in human data remains limited. In a clinical trial, the administration of choline supplements to asthma patients was found to considerably reduce immune inflammation levels and mitigate oxidative stress compared to standard medication treatment. This intervention also resulted in improved symptoms of airway obstruction.^14^ Additionally, it has been reported that serum choline levels are tellingly lower in asthma patients compared to healthy individuals, and this metabolic characteristic is related to a decline in lung function (including predicted forced expiratory volume in one second),^27^ suggesting that choline may feature in the pathogenesis of asthma. Our results indicated that increased dietary choline intake remarkably reduced the odds of asthma, addressing a gap in research on the linkage between dietary choline and asthma onset.

The protective impact of dietary choline on asthma might be attributed to two potential mechanisms. Firstly, it could be mediated through its anti‐inflammatory properties.^28^ Secondly, choline might engage in asthma pathogenesis via its involvement in metabolic pathways, leading to the generation of relevant metabolites. For instance, choline serves as a precursor for platelet‐activating factor (PAF) and acetylcholine.^9^ Notably, PAF has been established as a critical mediator in systemic allergic reactions.^29^ Its direct capacity to induce bronchial obstruction, along with its role as a driving factor for bronchial epithelial inflammation, contributes to heightened bronchial hyperresponsiveness,^30^, ^31^, ^32^ which compromises asthma control. Additionally, acetylcholine functions as a non‐selective muscarinic receptor agonist, directly affecting airway smooth muscle receptors to provoke bronchoconstriction. This principle underlies the development of acetylcholine challenge tests for asthma diagnosis.^33^, ^34^

Interestingly, our project revealed a dichotomy in the protective effect of dietary choline on asthma between adults (≥18 years) and children and adolescents (<18 years). While asthma can manifest at any age, it is noteworthy that adult‐onset asthma is often more closely associated with environmental and lifestyle factors rather than allergies and atopic conditions, in contrast to asthma diagnosed during childhood. The former is frequently linked to genetic predisposition and allergen sensitization.^35^, ^36^, ^37^ This article reports an average onset age of 11.33 years for childhood and adolescent asthma. Choline plays crucial and diverse roles in cellular maintenance and growth across all life stages, including functions in neural transmission, membrane synthesis, lipid transport, and participation in the one‐carbon metabolism pathway.^38^, ^39^, ^40^ Increasing dietary choline intake during pregnancy, lactation, and early life is vital for altering the structure and function of the brain and spinal cord. Moreover, it can, to some extent, reduce the likelihood of asthma in children and adolescents.^10^, ^38^, ^40^ Simultaneously, the GINA in 2021 emphasized that the “window of opportunity” for asthma prevention in children exists during prenatal and early life stages.^2^

Torén et al.‘s investigation into asthma projects and the onset year survey yielded a quite accurate average age of onset for adult asthma at 43.7 years.^41^ This study reported a similar onset age for adult asthma at 43.5 years, demonstrating a high degree of consistency. According to the latest research report from GBD 2019, the global age‐standardized prevalence of asthma in 2019 was 3415.53 cases per 100,000 population, indicating a substantial overall burden of asthma. The report also highlights that the highest impact on asthma‐related Disability‐Adjusted Life Years attributable to risk factors is from high BMI (peaking at ages 45–49), followed by occupational asthma agents and smoking.^42^ A meta‐analysis revealed a 50% increase in the incidence of asthma in overweight or obese individuals.^43^ This is primarily associated with asthma in obesity being related to an increase in fat factors, such as leptin, in visceral adipose tissue. Leptin and other fat factors may have a direct impact on the airways rather than enhancing airway inflammation, triggering obesity‐related asthma.^44^ In many industrialized nations, occupational asthma is the most common type of adult‐onset asthma, with at least 9%–15% of adult asthma cases associated with it. Recent data suggest that 25% or more of asthma cases may be linked to occupational exposure.^45^, ^46^ Regarding lifestyle factors, active and passive smoking, along with continuous exposure to airborne allergens and smoking, may have cumulative or synergistic effects.^35^ Therefore, dietary choline intake, as a lifestyle factor, exhibiting a significant protective effect in adult asthma patients appears reasonable.

Choline, as an essential nutrient, plays a critical role throughout the lifecycle. Therefore, it is necessary to supplement an adequate dietary choline intake daily to meet physiological functions related to brain development, liver, and cardiovascular health. The Adequate Intake (AI) recommended by the Food and Nutrition Board of the National Academy of Sciences and Medicine, and the Institute of Medicine suggests an average daily intake of approximately 7.5 mg of choline per kilogram of body weight for adults. The AI is set at 550 mg/d for males and 425 mg/d for females, based on reference weights of 76 kg for males and 59 kg for females. However, considering the needs during pregnancy and lactation, the intake of choline is higher during these periods. The AI for choline during pregnancy is set at 450 mg/d, while for infants aged 0–6 months, the AI is 125 mg/d, and for infants aged 7–12 months, it is set at 150 mg/d using weight‐based calculations. For children and adolescents, the choline AI is calculated using the formula: AI = AI adult (weight child/weight adult)^0.75^ (1 + growth factor). The growth factors for children are 0.30 between 7 months and 3 years, 0.15 between 4 and 13 years, 0.15 for males between 14 and 18 years, and 0.00 for females between 14 and 18 years.^47^

BMI, commonly used to assess body weight status, categorizes individuals with a BMI between 25 and 30 kg/m^2^ as overweight and those with a BMI >30 kg/m^2^ as obese.^48^ Our study demonstrated that in the population with a BMI >25 kg/m^2^, an increased dietary choline intake considerably lowered the odds of asthma. The reciprocal association between obesity and asthma has been established,^49^ presenting a challenge for researchers. BMI is positively associated with poorly controlled asthma and worsening symptoms in children and adolescents, as well as increased acute exacerbations in adult asthma patients.^50^, ^51^ Obese individuals with asthma tend to experience persistent symptoms, more frequent emergency visits, longer hospital stays, and higher medical costs.^52^, ^53^, ^54^ This is possibly due to the heightened inflammatory state in obesity, with adipose tissue releasing pro‐inflammatory molecules such as IL‐6, TNF‐α, C‐reactive protein, and leptin, which drive inflammatory responses.^55^ Moreover, obesity is linked with systemic leukotriene inflammation in asthma individuals, with leukotrienes playing a key role in bronchoconstriction.^56^, ^57^ Existing evidence indicates that proactive weight management significantly improves asthma symptoms and quality of life in obese asthma patients,^58^ achieved through dietary interventions, physical activity, or weight loss surgery.^59^ Even modest weight loss can confer clinical benefits for asthma patients,^60^ underscoring the importance of encouraging active weight management for overweight and obese (BMI >25 kg/m^2^) individuals with asthma. In light of our research findings, the supplementation of dietary choline could be a beneficial approach, especially in the overweight and obese populations, to potentially prevent the onset of asthma.

This work boasted several strengths, including its utilization of a large sample size across multiple survey cycles contributing to its representative nature. However, it is important to acknowledge certain limitations. Firstly, the cross‐sectional nature of the NHANES database precluded temporal inference, hindering the establishment of a causal relationship between dietary choline intake and asthma, necessitating further research. Moreover, the data in this investigation largely relied on survey reports from participants' recall, introducing potential information bias. Furthermore, there are numerous potential confounding factors for asthma. Although we included as many relevant covariates as possible in our model, we cannot completely rule out the influence of other potential confounders, which may affect our understanding of the relationship between dietary choline intake and the odds of asthma. Additionally, different statistical methods and model choices may also influence the results. Despite these limitations, our study still uncovered a noteworthy inverse relationship between increased dietary choline intake and the odds of asthma, offering valuable insights into the field.

## AUTHOR CONTRIBUTIONS

Conceptualization: Jiaqiang Shi. Data curation: Yuming Lin, Yingxiu Jiang. Formal Analysis: Yingxiu Jiang. Investigation: Guoguo Qiu, Fanghua Jian. Methodology: Guoguo Qiu, ShiHao Zhang. Project administration: Jiaqiang Shi. Resources: Wei Lin, ShiHao Zhang. Software: Wei Lin. Supervision: Wei Lin. Validation: ShiHao Zhang. Visualization: Yingxiu Jiang. Writing – original draft: Jiaqiang Shi, Yuming Lin. Writing – review & editing: Yingxiu Jiang, Guoguo Qiu, ShiHao Zhang.

## CONFLICT OF INTEREST STATEMENT

The authors report no conflicts of interest.

## Data Availability

The datasets generated and analyzed during the current study are not publicly available but are available from the corresponding author on reasonable request.
